# Characterization of *Escherichia coli* Strains for Novel Production of *Plasmodium ovale* Lactate Dehydrogenase

**DOI:** 10.3390/microorganisms12050876

**Published:** 2024-04-27

**Authors:** Jae-Won Choi, Sang-Oh Ha, Yeon-Jun Kim, Jun-Seop Shin, Min-Ji Choi, Si-Eun Yu, Junghun Han, Eun-Ji Park, Kyoung Sik Park, Jung Hoon Kang

**Affiliations:** 1 Department of Biopharmaceutical Sciences, Cheongju University, Cheongju 28160, Republic of Korea; 2 Department of Biomedical Science, Cheongju University, Cheongju 28160, Republic of Korea; 3 Department of Pharmaceutical and Biopharmaceutical Sciences, Cheongju University, Cheongju 28160, Republic of Korea

**Keywords:** malaria, *Plasmodium ovale*, lactate dehydrogenase, *Escherichia coli*

## Abstract

Malaria is one of the most prevalent diseases worldwide with high incidence and mortality. Among the five species that can infect humans, *Plasmodium ovale* morphologically resembles *Plasmodium vivax*, resulting in misidentification and confusion in diagnosis, and is responsible for malarial disease relapse due to the formation of hypnozoites. *P. ovale* receives relatively less attention compared to other major parasites, such as *P. falciparum* and *P. vivax*, primarily due to its lower pathogenicity, mortality rates, and prevalence rates. To efficiently produce lactate dehydrogenase (LDH), a major target for diagnosing malaria, this study used three *Escherichia coli* strains, BL21(DE3), BL21(DE3)pLysS, and Rosetta(DE3), commonly used for recombinant protein production. These strains were characterized to select the optimal strain for *P. ovale* LDH (PoLDH) production. Gene cloning for recombinant PoLDH production and transformation of the three strains for protein expression were performed. The optimal PoLDH overexpression and washing buffer conditions in nickel-based affinity chromatography were established to ensure high-purity PoLDH. The yields of PoLDH expressed by the three strains were as follows: BL21(DE3), 7.6 mg/L; BL21(DE3)pLysS, 7.4 mg/L; and Rosetta(DE3), 9.5 mg/L. These findings are expected to be highly useful for PoLDH-specific diagnosis and development of antimalarial therapeutics.

## 1. Introduction

Malaria is a febrile, infectious disease caused by *Plasmodium* spp. transmitted through the bite of female mosquitoes of the genus *Anopheles* that mediate parasite entry into the human body [1]. According to the World Malaria Report 2023, an estimated 250 million new cases of malaria occurred in 2022 alone, causing approximately 600,000 deaths [2]. Upon onset of malaria caused by *Plasmodium* infection, patients exhibit typical symptoms, including fever, headache, myalgia, nausea, and vomiting. When patients progress to severe malaria, they develop anemia, organ failure, including liver or renal tissue, and decreased consciousness [3,4,5]. *Plasmodium* parasite exhibits a complex lifecycle, undergoing multiple stages and affecting multiple tissues, requiring extensive asexual replication in the liver cells and erythrocytes of the vertebrate host and in the hemocoel of the insect vector, followed by a single round of sexual reproduction producing hundreds and thousands of daughter cells [6]. To date, more than 200 species of *Plasmodium* have been identified, of which only five species are responsible for causing malaria in humans: *P. falciparum*, *P. vivax*, *P. ovale*, *P. malariae*, and *P. knowlesi* [7].

*P. ovale* is the main species of this study and forms hypnozoites in its lifecycle, as in the case of *P. vivax*, remaining dormant for weeks or even months and then reactivating to cause malaria relapse [8]. In addition, the widespread misidentification of *P. ovale* as *P. vivax* by microscopy commonly occurs [9]. Due to the chloroquine resistance of *P. falciparum*, the dominant species [10], artemisinin-based therapies [11] or proguanil [12] are used for treatment. In the case of *P. ovale*, the approach to treatment differs due to the use of primaquine [13] or tafenoquine [14]. While *P. falciparum* and *P. vivax* are the most studied species since they account for the highest proportion of malaria cases worldwide, *P. ovale* is considerably understudied owing to its relatively low pathogenicity and prevalence [15,16]. Nevertheless, *P. ovale* infections are frequently reported in East Asia, Southeast Asia, and Southwest Africa, warranting investigation to gain an in-depth understanding of *P. ovale* [17,18].

The main proteins that play a role as biomarkers for malaria research include histidine-rich protein-2 (HRP-2) [19], merozoite surface protein (MSP) [20], lactate dehydrogenase (LDH) [21,22], glutamate dehydrogenase (GDH) [23], and aldolase [24], of which LDH is the most commonly used biomarker. LDH is a glycolytic enzyme that catalyzes the reversible conversion of lactate to pyruvate by removing hydrogen from lactic acid. It functions by forming a tetramer and each monomer (with a molecular weight of approximately ~34 kDa) is folded into two domains. The *Plasmodium* parasite is a scavenger that continuously aims to invade erythrocytes (red blood cells) in the human body. It increases the glycolytic rate by over 100-fold when it successfully invades red blood cells [25]. A rapid increase in the glycolytic rate indicates that the glycolytic pathway that breaks down glucose is active, representing a rapid increase in LDH, which is involved in the final step of glycolysis [26]. *Plasmodium* is abundant in erythrocytes at the early trophozoite stage, wherein asexual reproduction occurs, and *Plasmodium* displays its maximum metabolic activity. Therefore, *Plasmodium* spp. LDH has been widely studied as a useful biomarker and therapeutic target for diagnosing and treating malaria. However, although the *P. falciparum* and *P. vivax LDH* genes show high homology with *P. ovale LDH* (*PoLDH*) gene sequences, differences in amino acid sequences necessitate research explicitly focusing on PoLDH.

For the expression of eukaryotic proteins, recombinant protein production using the expression systems of mammalian or insect cells has the advantage of remaining close to the native form. However, using these expression systems has the disadvantages of being time-consuming, labor-intensive, and costly. In response to these limitations, bacterial expression systems have been widely used to express and purify recombinant proteins [27,28,29]. Currently, research on the expression of eukaryotic proteins is actively conducted using *Escherichia coli* (*E. coli*) strains, including DH5α [30,31], BL21(DE3) [32], BL21(DE3)pLysS [33], and Rosetta(DE3) [34]. *E. coli* was selected as a suitable bacterial expression system owing to its faster growth rate relative to that of animal cells while requiring much less labor and cost inputs. Therefore, we aimed to establish methods and conditions to enable the mass production of PoLDH by identifying the optimal expression conditions and *E. coli* strains for producing PoLDH.

## 2. Materials and Methods

### 2.1. Cloning P. ovale LDH Gene

A *PoLDH* gene was designed based on the nucleotide sequence obtained from the National Center for Biotechnology Information (accession No. KM226655.1) with a *Bam*HI site at the 5′-end and an *Xho*I site at the 3′-end and synthesized into a pUC-IDT (Integrated DNA Technologies (IDT), Coralville, IA, USA) containing the ampicillin-resistance gene. The synthesized pUC-IDT-*PoLDH* plasmid was PCR-amplified to produce a large amount of *PoLDH.* The forward and reverse primers used for PCR amplification were 5′-GGA TCC ATT GTG CTC GTC GG-3′ and 5′-CTC GAG AAT GAG CGC CTT CAT-3′, respectively. The detailed PCR cycle conditions were as follows: initial denaturation at 95 °C for 10 min for one cycle, followed by 35 cycles of denaturation at 95 °C for 30 s, annealing at 60 °C for 45 s, and extension at 72 °C for 1 min, and then one final extension cycle at 72 °C for 5 min. Subsequently, *PoLDH* was purified, quantified, and prepared as insert DNA to construct recombinant plasmids. The purified PCR product and expression vector pET-21a(+) were digested using *Bam*HI (20 units) and *Xho*I (20 units) (New England Biolabs (NEB), Ipswich, MA, USA). The digested DNA fragments were separated on agarose gel and extracted using Expin^TM^ Combo GP (GeneAll Biotechnology, Seoul, Republic of Korea). The *PoLDH* and pET-21a(+) fragments extracted from the agarose gel were ligated using T4 DNA ligase (1 unit; NEB) by reacting at room temperature for 16 h. The recombinant pET-21a(+)-*PoLDH* was introduced into the DH5α strain for stable mass production. DH5α competent cells (Enzynomics, Daejeon, Republic of Korea) and the ligation product were mixed and stabilized by incubation on ice. Subsequently, the heat was applied at 42 °C for 1 min, and the recombinant plasmid was introduced into the competent cells and underwent stabilization on ice. Next, super optimal broth with catabolite repression was added, and the mixture was cultured at 200 rpm for 1 h at 37 °C. The cultured cells were spread on Luria-Bertani (LB) agar plate containing ampicillin at a concentration of 100 μg/mL and incubated overnight at 37 °C. A transformed DH5α cell line was constructed from the single colony formed. To confirm the successful insertion of the recombinant plasmid into the strain, plasmids extracted from the transformed strain were subjected to PCR analysis and digestion with restriction enzymes. Finally, the successful production of recombinant pET-21a(+)-*PoLDH* was verified by agarose gel electrophoresis. In addition, the cDNA sequence was analyzed using specific primers to T7 promoter and T7 terminator on a 3730xl DNA analyzer (Applied Biosystems, Waltham, MA, USA).

### 2.2. Selecting the Optimal Strain for PoLDH Expression

#### 2.2.1. Transformation of *E. coli* Strains

To determine the optimal strain for PoLDH protein expression, three *E. coli* strains were used: BL21(DE3), BL21(DE3)pLysS, and Rosetta(DE3). *E. coli* BL21(DE3) and Rosetta(DE3) competent cells were purchased from Enzynomics. *E. coli* BL21(DE3)pLysS competent cells were prepared using the calcium chloride (CaCl_2_) method. BL21(DE3)pLysS were added to fresh (no antibiotics) LB broth and shake incubated at 37 °C and 200 rpm until the optical density at 600 nm (OD_600_), measured using a UV-Vis spectrophotometer (OPTIZEN, Daejeon, Republic of Korea), reached 0.5. The culture was then incubated on ice and centrifuged at 4 °C and 4500 rpm for 10 min. After discarding the supernatant, the pellet was resuspended by adding 0.1 M CaCl_2_ in the same volume as the culture. After centrifugation, the supernatant was discarded again, and competent cells were produced by mixing 50% glycerol and 0.1 M CaCl_2_ solution in a 1:1 ratio and combining the pellets. The prepared competent cells were divided into aliquots and stored in a deep freezer. In the same procedure as that for transforming the DH5α strain, each strain was transformed by treatment with 0.1 ng pET-21a(+)-*PoLDH* plasmid to prepare strains for recombinant PoLDH expression.

#### 2.2.2. Comparison of PoLDH Expression Patterns in the *E. coli* Strains

The PoLDH overexpression saturation time points for each strain cultured were determined with different concentrations of isopropyl β-D-1-thiogalactopyranoside (IPTG): 0, 0.001, 0.005, 0.01, 0.05, 0.1, 0.5, and 1 mM. The three transformed strains were incubated in LB broth containing ampicillin until the OD_600_ reached 0.7. Thereafter, IPTG was added to the cell culture at the concentrations indicated above, and the mixture was incubated at 18 °C and 200 rpm for 16 h. The culture was centrifuged at 13,000 rpm, after which the supernatant was discarded, and the remaining pellet was resuspended in phosphate-buffered saline (PBS) and mixed with 4× sample buffer (250 mM Tris-HCl (pH 6.8), 8% SDS, 40% glycerol, 8% β-mercaptoethanol, and 0.02% bromophenol blue) to perform sodium dodecyl sulfate-polyacrylamide gel electrophoresis (SDS-PAGE) analysis. Subsequently, the prepared samples were separated into a 12% polyacrylamide gel. After electrophoresis, the gel was stained with Coomassie Brilliant Blue R-250 (CBB) to compare the PoLDH saturation point by IPTG concentration for each strain. Second, the PoLDH expression patterns were compared for each strain according to the incubation time of the strains with 0.1 mM IPTG. The three transformed strains were incubated in LB broth containing ampicillin until the OD_600_ reached 0.7. Subsequently, IPTG was added to a final concentration of 0.1 mM, and samples were obtained every hour while incubating at 18 °C and 200 rpm for a maximum of 16 h. The obtained culture was centrifuged at 13,000 rpm. After discarding the supernatant, the remaining pellet was resuspended in PBS and mixed with 4× sample buffer to prepare for analysis through SDS-PAGE. The prepared samples were similarly separated and electrophoresed into a 12% polyacrylamide gel, after which the gel was stained with CBB to compare the PoLDH expression saturation time points for each strain according to IPTG induction time.

### 2.3. Establishing Optimized Operating Conditions for PoLDH Purification

#### 2.3.1. Conditions for Purifying PoLDH Protein

To establish operating conditions for purification to obtain high-purity and high-yield PoLDH, PoLDH overexpression was performed using the Rosetta(DE3) strain. The transformed Rosetta(DE3) strain was incubated in LB broth containing ampicillin until the OD_600_ reached 0.7. The bacterial culture was incubated with 0.1 mM IPTG at 18 °C and 200 rpm for 16 h and then centrifuged at 7500 rpm for 45 min at 4 °C. Thereafter, equilibrium buffer (pH 8.0; 500 mM NaCl, 50 mM KH_2_PO_4_, 5 mM imidazole), 100× Xpert Protease Inhibitor (GenDEPOT, Barker, TX, USA), and lysozyme (Sigma-Aldrich, St. Louis, MO, USA) were added to the resulting pellet and thoroughly mixed. The mixture was lysed using a sonicator (Sonics & Materials, Newtown, CT, USA) for 30 min (10 s of sonication followed by 20 s rest) at 30% amplitude. Subsequently, the lysate was centrifuged at 9000 rpm for 30 min at 4 °C, after which the supernatant was prepared as a sample for affinity chromatography by passing through a 0.22 μm syringe filter (GVS, Bologna, Italy). His·Bind^®^ nickel-nitrilotriacetic acid (Ni-NTA) resin (Merck Millipore, Burlington, MA, USA) was packed into an empty column up to a bed volume of 2 mL. The resin was equilibrated with an equilibrium buffer. Next, all prepared protein samples were loaded onto the column pre-packed with the resin. A washing buffer was prepared using 500 mM NaCl and 50 mM KH_2_PO_4_ with four different imidazole concentrations (pH 8.0; 10, 20, 30, and 40 mM). Thereafter, washing buffers with four different imidazole concentrations were consecutively loaded onto each resin to obtain washing fractions that passed through the resin. As the last step, 20 mL of elution buffer (pH 8.0; 500 mM NaCl, 50 mM KH_2_PO_4_, and 500 mM imidazole) was loaded onto the resin to obtain a 6× His-tagged PoLDH elution fraction. Fractions obtained during purification for each imidazole concentration of the washing buffer were compared through SDS-PAGE analysis. Furthermore, the purity of the purified PoLDH was compared by calculating the area fraction of the second elution fraction on each gel using ImageJ software (ver. 1.54f, National Institutes of Health, Bethesda, MD, USA).

#### 2.3.2. Comparison of PoLDH Purification Yield for Each Strain

The three *E. coli* strains—BL21(DE3), BL21(DE3)pLysS, and Rosetta(DE3)—were incubated in LB broth containing ampicillin until the OD_600_ reached 0.7. Bacterial cultures were incubated with 0.1 mM IPTG for 16 h at 18 °C and 200 rpm. The three strains were lysed through sonication under the same conditions. Afterward, the supernatant obtained by centrifuging the lysate was pooled and subjected to filtration. Three protein samples from the three strains were purified using Ni-NTA resin. For PoLDH purification, equilibrium buffer, washing buffer (pH 8.0; 500 mM NaCl, 50 mM KH_2_PO_4_, and 40 mM imidazole), and elution buffer (pH 8.0; 500 mM NaCl, 50 mM KH_2_PO_4_, and 500 mM imidazole) were used. A series of dialysis steps were conducted to replace the elution buffer; all dialysis steps were performed at 4 °C to prevent protein denaturation. The first round of dialysis was performed overnight in PBS (pH 7.4) containing 50 mM imidazole, and the second round was performed with PBS without imidazole. Finally, PBS was replaced once more, and a third round of dialysis was performed. To remove remaining impurities, filtration was performed through a 0.22 μm syringe filter to finally obtain PoLDH for use in subsequent experiments. PoLDH expressed and purified from each of the three strains was quantified by measuring absorbance at 562 nm following a bicinchoninic acid (BCA) protein assay (Thermo Fisher Scientific, Waltham, MA, USA). The total amount of each PoLDH was calculated proportionately to the culture volume, and then the yield for each strain was compared.

### 2.4. Analysis of Tetramer Formation and Enzyme Activity in the Purified PoLDH

To determine the presence of monomer formation, 2 µg of PoLDH was mixed with 4× sample buffer, boiled at 100 °C, and separated into a 12% polyacrylamide gel. The molecular weight and purity of the purified PoLDH were assessed through CBB staining by comparison with a protein size marker. To confirm the tetramer formation, the active form of PoLDH, 2 µg of PoLDH was mixed with 4× sample buffer without SDS and separated into a 10% separating gel without SDS. The molecular weight of PoLDH with tetramer formation was assessed through CBB staining by comparison with a protein size marker. A colorimetric LDH assay kit (Abcam, Cambridge, UK) was used to analyze the enzyme activity of the purified PoLDH. To each 96-well plate, 50 µL of NADH diluted to 0, 2.5, 5.0, 7.5, 10.0, and 12.5 nmole was loaded. At this time, 48 μL of LDH assay buffer and 2 μL of substrate mix were mixed, and the absorbance was measured at 450 nm to obtain a standard curve according to NADH concentration. Next, the purified PoLDH was diluted with the LDH assay buffer to final concentrations of 0, 0.02, 0.03, 0.06, 0.13, 0.25, and 0.50 nM, and 50 µL of PoLDH was loaded into a 96-well plate. Subsequently, 48 μL of LDH assay buffer and 2 μL of substrate mix were mixed and reacted at 37 °C in the absence of light. Absorbance was measured at 450 nm using a microplate reader (BioTek, Winooski, VT, USA) at 10 min and 30 min time points after the reaction.

## 3. Results and Discussion

### 3.1. PoLDH Recombinant Plasmid Construction and Transformation of E. coli Strains for Protein Expression

**Figure 1:**
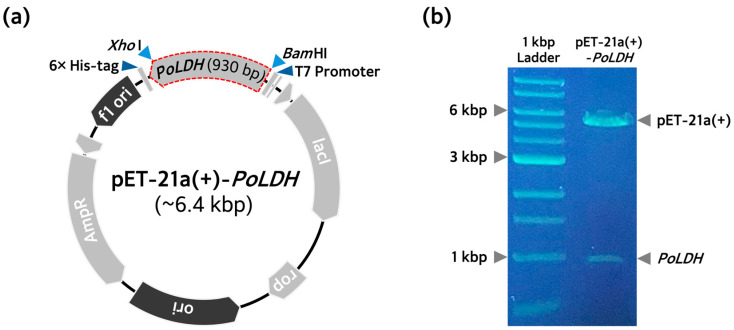
Plasmid construction for recombinant PoLDH production. (**a**) Schematic diagram of plasmid construction with *PoLDH* cDNA. The *PoLDH* gene obtained through PCR from pUC-IDT-*PoLDH* was introduced through the multiple cloning site of the expression vector pET-21a(+). Through digestion with restriction enzymes (*Bam*HI and *Xho*I) and ligation with T4 ligase, recombinant pET-21a(+)-*PoLDH* was constructed to produce recombinant PoLDH. (**b**) Identification of purified recombinant pET-21a(+)-*PoLDH* extracted from the DH5α strain. An agarose gel image of the purified plasmid extracted from the DH5α strain after digestion with both restriction enzymes (*Bam*HI and *Xho*I). Gel electrophoresis was performed on a 0.7% agarose gel.

For expression of the PoLDH, we successfully constructed the pET-21a(+)-*PoLDH* recombinant plasmid (Figure 1a). The T7 promoter in pET-21a(+) plasmid facilitates the overexpression of proteins from various organisms and has the advantage of allowing protein expression in hosts other than *E. coli* [35]. To confirm that the pET-21a(+)-*PoLDH* recombinant plasmid was correctly constructed, they were subjected to digestion with *Bam*HI and *Xho*I restriction enzymes. When the 5′- and 3′-ends were cleaved with *Bam*HI and *Xho*I restriction enzymes, bands for the pET-21a(+) vector and *PoLDH* gene formed at ~5 and ~1 kbp, respectively (Figure 1b). Thus, the formation of these bands confirmed that the pET-21a(+)-*PoLDH* recombinant plasmid was correctly constructed. Next, the constructed recombinant plasmids were introduced into the three *E*. *coli* strains, BL21(DE3), BL21(DE3)pLysS, and Rosetta(DE3), which were used to overexpress PoLDH recombinant proteins, and these strains were transformed.

### 3.2. Comparison of PoLDH Expression Pattern and Overexpression Optimization for Different E. coli Strains 

**Figure 2:**
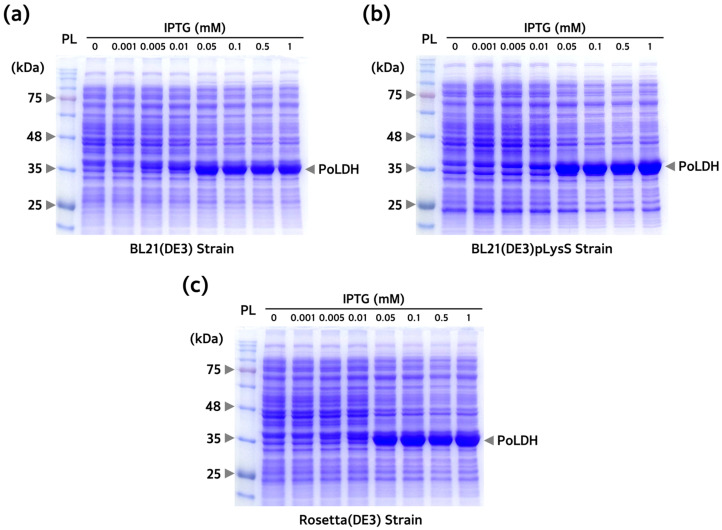
Optimization of IPTG concentration for PoLDH expression. Sodium dodecyl sulfate-polyacrylamide gel electrophoresis (SDS-PAGE) images of PoLDH expressed from (**a**) BL21(DE3), (**b**) BL21(DE3)pLysS, and (**c**) Rosetta(DE3) according to IPTG concentration. All three experiments were conducted at IPTG concentrations of 0, 0.001, 0.005, 0.01, 0.05, 0.1, 0.5, and 1 mM and incubation conditions at 18 °C and 200 rpm for 16 h. Arrows on the right side of the gel indicate overexpressed PoLDH. PL, protein ladder.

**Figure 3:**
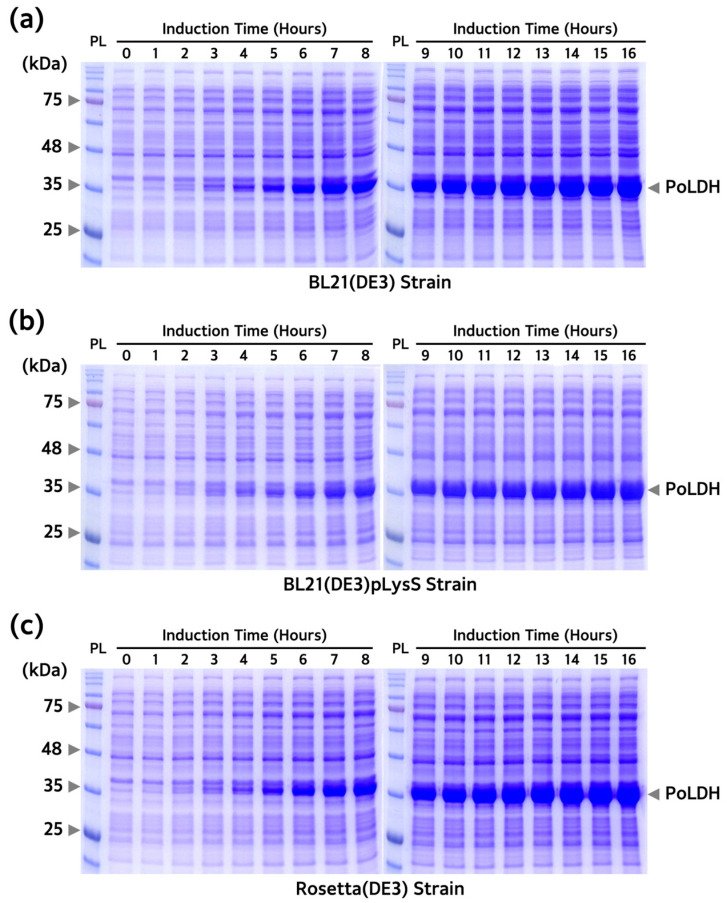
Expression patterns of PoLDH according to IPTG induction time for the three *E. coli* strains. SDS-PAGE images of PoLDH expressed from (**a**) BL21(DE3), (**b**) BL21(DE3)pLysS, and (**c**) Rosetta(DE3) according to IPTG induction time. All three experiments were conducted with an IPTG concentration of 0.1 mM and incubation conditions of 18 °C and 200 rpm for 16 h. Arrows on the right side of the gel indicate overexpressed PoLDH. PL, protein ladder.

To overexpress the PoLDH recombinant protein, differences in protein expression between BL21(DE3), BL21(DE3)pLysS, and Rosetta(DE3) were examined. pET-21a(+)-*PoLDH* plasmids were introduced into all strains. First, each culture was treated at different IPTG concentrations, followed by induction at 18 °C for 16 h to optimize the concentration of IPTG used for protein overexpression. Induction was performed at low temperatures since it has a positive effect on increasing protein yields and correct folding. A previous study also showed that successful production of recombinant PvLDH was achieved at 18 °C [36]. Successful overexpression of a protein presumed to be PoLDH was achieved at ~35 kDa in all three strains and that saturation started from an IPTG concentration of 0.1 mM (Figure 2). Subsequently, IPTG was added to the culture at a concentration of 0.1 mM containing the three transformed strains and incubated at 18 °C for 16 h. The strains were harvested every hour and separated by SDS-PAGE to confirm PoLDH overexpression in the strains after incubation. Protein saturation started from 12 h (Figure 3). In addition, elevated Rosetta(DE3) expression levels were confirmed by SDS-PAGE loaded with the same number of bacteria. Decreasing the IPTG concentration used for overexpression of recombinant PoLDH leads to increased cost-effectiveness of the experimental procedure. Furthermore, lowering the overexpression temperature enhances protein solubility, providing additional advantages. Therefore, incubation at 18 °C for 16 h and 0.1 mM IPTG were considered optimal conditions for PoLDH overexpression.

### 3.3. Optimization of Buffer Conditions for Isolating and Purifying PoLDH

**Figure 4:**
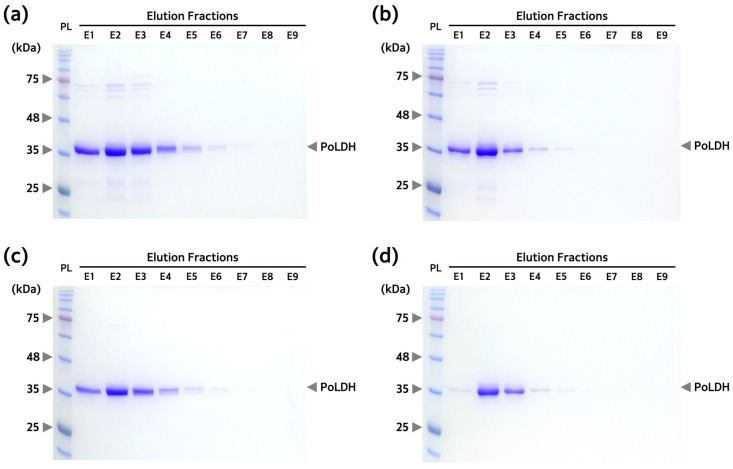
Elution patterns of PoLDH according to the concentration of imidazole in the washing buffer. SDS-PAGE images of the elution fractions with (**a**) 10 mM, (**b**) 20 mM, (**c**) 30 mM, and (**d**) 40 mM imidazole in washing buffer. Otherwise, all experiments were performed under the same conditions. The eluted PoLDH was expressed from the Rosetta(DE3) strain. SDS-PAGE was performed on a 12% polyacrylamide gel. Arrows on the right side of the gel indicate PoLDH present in the elution fractions. PL, protein ladder.

To establish the optimal conditions for isolating and purifying PoLDH, Rosetta(DE3) was used since it showed the highest PoLDH expression levels per number of bacteria. Non-specific proteins other than the target protein to which the 6× His-tag is attached contain histidine residues and have some binding force. Therefore, the imidazole concentration of the washing buffer used to remove non-specific, unbound proteins is important to the purification process. If the optimal imidazole concentration is not used, non-specific proteins may be detected together with the target protein, or the target protein may be washed out. Therefore, we used four washing buffers containing 10, 20, 30, and 40 mM imidazole to find the optimal concentration of imidazole in the washing buffer since non-specific protein binding occurs. There was a decrease in non-specific protein bands with increasing imidazole concentration in the washing buffer. To examine whether the washing buffer properly removed non-specific proteins, the ratio of PoLDH to the total area of the elution 2 lane was calculated using ImageJ software. The results for the calculated area fraction were 74.0% for an imidazole concentration of 10 mM, 81.0% for 20 mM, 98.3% for 30 mM, and 99.9% for 40 mM (Figure 4). Therefore, the optimal imidazole concentration in the washing buffer for PoLDH purification is 40 mM.

### 3.4. Comparison of PoLDH Purification and Yields between Different E. coli Strains

**Figure 5:**
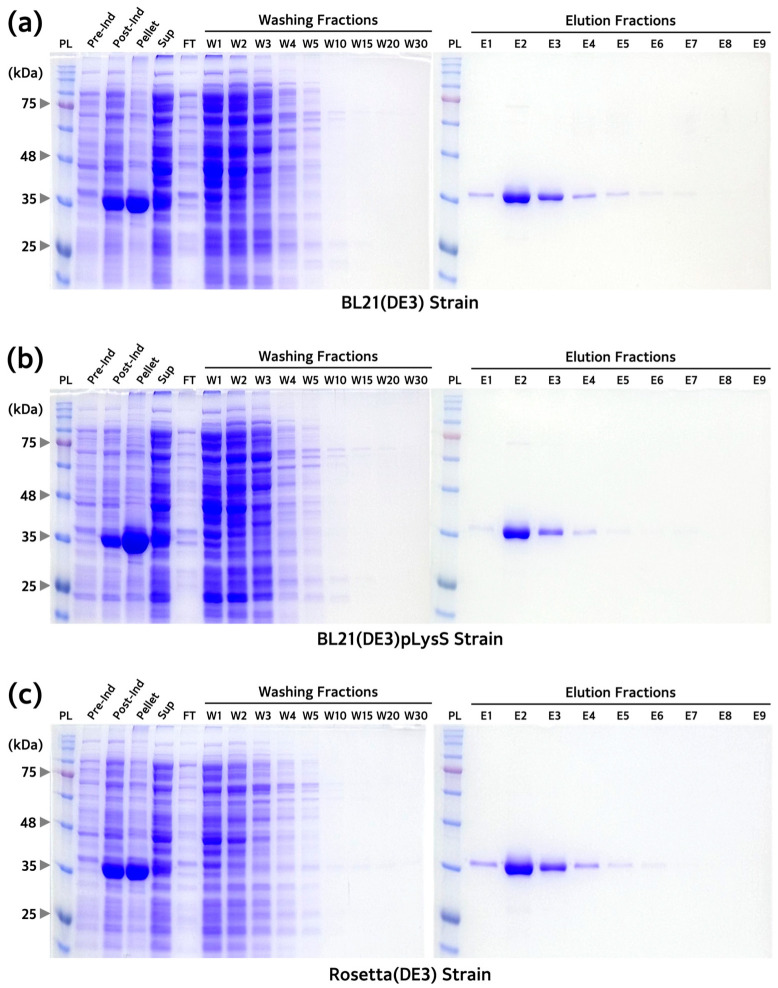
Analysis of fractions obtained from the purification process of PoLDH expressed from the three *E. coli* strains. SDS-PAGE images of fractions from the purification of PoLDH expressed from (**a**) BL21(DE3), (**b**) BL21(DE3)pLysS, and (**c**) Rosetta(DE3). All experiments were performed under the same conditions. SDS-PAGE was performed on a 12% polyacrylamide gel. PL, protein ladder; Pre-Ind, pre-induction; Post-Ind, post-induction; Sup, supernatant; FT, flow-through.

After establishing the optimal PoLDH overexpression and washing buffer for purification conditions, the yield among the three *E*. *coli* strains was compared through the purification of the expressed PoLDH. While more PoLDH was observed in the pellets compared to the supernatants after the sonication of each strain, purification was carried out using the supernatant due to the significant presence of PoLDH. This choice was made to avoid the need for extensive chaotropic reagents, and the time required for solubilizing the pellets. When the flow-through fraction passing through the Ni-NTA resin during purification was analyzed using SDS-PAGE, the results confirmed that binding to resin was properly achieved, considering only a small number of PoLDH bands. Moreover, non-specific proteins, excluding PoLDH, were removed through repeated washing from the pre-washing state to washing fraction 30 (W30, described in Figure 5). In addition, 6× His-tagged PoLDH was eluted when the elution buffer (containing 500 mM imidazole) flowed through the resin (Figure 5). After purification, proteins were quantified using the BCA protein assay method to determine the yield of each strain. The yields of PoLDH obtained using BL21(DE3), BL21(DE3)pLysS, and Rosetta(DE3) were 7.6, 7.4, and 9.5 mg/L, respectively.

Among the three strains used in this study, BL21(DE3) displayed high protein expression only when using the T7 promoter as in the case of the pET vector; however, the strain has leaky expression under the condition of no IPTG, indicating that basal protein expression that is not completely controlled by *lac*I [29]. BL21(DE3)pLysS expressed proteins only after adding IPTG because of its own lysozyme suppressing T7 RNA polymerase (no leaky expression), although it had reduced protein expression owing to suppression by lysozyme compared with BL21(DE3) [29]. Finally, Rosetta(DE3), a strain derived from BL21(DE3) increases the expression of eukaryotic proteins by including codons (AGA, AGG, AUA, CCC, CUA, and GGA) rarely used in *E. coli* [34]. Among the three strains used in the present study, BL21(DE3) and BL21(DE3)pLysS showed similar levels of yield, but Rosetta(DE3) showed a 25.0–28.4% greater yield compared with that from these other strains. Therefore, Rosetta(DE3) was determined to be the optimal strain for PoLDH expression. However, the yield of PoLDH was only 30.6% (9.5 mg/L) of that of PvLDH (31.0 mg/L) reported in a previous study [36], despite applying optimal buffer conditions. The yield of PoLDH may be directly related to solubility. Firstly, the amino acid sequence itself can affect solubility due to conformational similarity. We input the amino acid sequences of PoLDH and PvLDH into ‘Protein-Sol’, a sequence-based protein solubility prediction tool [37]. Although PoLDH and PvLDH show over 98% similarity, the solubility of PoLDH was calculated as 0.537, while PvLDH was calculated as 0.539. There was a slight difference, but the solubility of PoLDH was lower than that of PvLDH. Secondly, we utilized ‘CamSol Intrinsic pH-dependent’, a tool capable of calculating protein solubility by considering factors, such as the buffer pH, and amino acid sequence [38]. The solubility of PoLDH at the pH 8.0 of the buffer used in our purification process was determined to be −0.073793, while PvLDH was 0.284086. Additionally, within the widely utilized pH range of 7.0 to 8.0 in protein work, the solubility of PoLDH was consistently lower than that of PvLDH. This also demonstrates consistency with our experimental results.

### 3.5. Confirmation of Tetramer Formation in the Purified PoLDH

**Figure 6:**
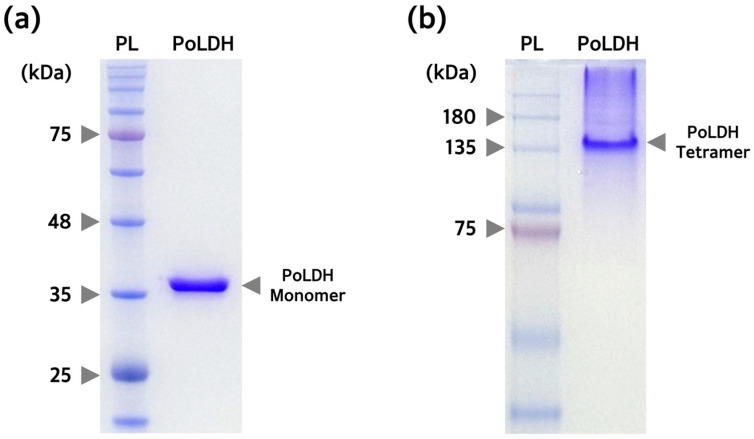
PAGE analysis to confirm the purity and tetramer formation of purified PoLDH. (**a**) SDS-PAGE image confirming the monomer formation and purity of PoLDH. SDS-PAGE was performed on a 12% polyacrylamide gel. (**b**) Native PAGE image confirming the tetramer formation of PoLDH. The purified PoLDH was mixed with an SDS-free sample buffer and loaded onto an SDS-free 10% polyacrylamide gel. PL, protein ladder.

It is well documented that LDH forms tetramers and has enzyme activity, as does PoLDH. Therefore, to determine whether the purified PoLDH is properly folded to form tetramers in Rosetta(DE3), SDS-PAGE was performed on PoLDH under reducing and non-reducing conditions. A single PoLDH monomer band was observed at ~35 kDa when PoLDH was separated under reducing conditions (Figure 6a). A single PoLDH tetramer band was identified between ~135 and ~180 kDa when PoLDH was separated under non-reducing conditions through native PAGE (Figure 6b). Therefore, PoLDH expressed from the Rosetta(DE3) strain could be easily purified in a soluble form and folded into a three-dimensional structure.

### 3.6. Analysis of Enzyme Activity of the Purified PoLDH

**Figure 7:**
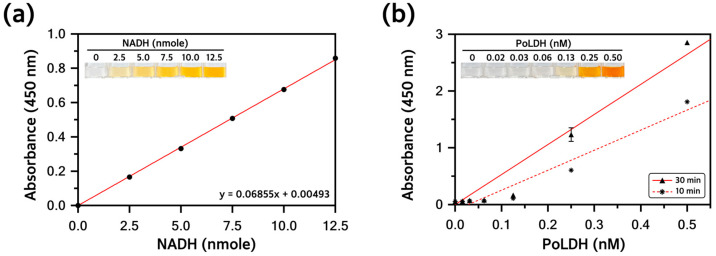
Analysis of the purified PoLDH enzyme activity. (**a**) Standard curve of different amounts of NADH (0, 2.5, 5.0, 7.5, 10.0, and 12.5 nmole). The upper left inset image shows color change with the amount of NADH. (**b**) Absorbance measurement for each time unit (10 and 30 min) at different final concentrations of PoLDH (0, 0.02, 0.03, 0.06, 0.13, 0.25, and 0.50 nM). Absorbance was measured at a wavelength of 450 nm, 10 min and 30 min after the reaction at 37 °C.

A colorimetric LDH activity assay was conducted to determine whether the purified PoLDH exhibited enzyme activity. Lactic acid is converted to pyruvic acid by LDH and NAD^+^ is reduced to NADH at the same time. The enzyme activity of purified PoLDH was determined considering the production of NADH, which appears as a yellow complex compound with a maximum absorbance at a wavelength of 450 nm. First, a standard curve with the equation *y* = 0.06855*x* + 0.00493 was derived from the prepared amounts of NADH (Figure 7a). The amount of NADH produced by PoLDH for 20 min was calculated using different PoLDH concentrations and substrate mixtures, with enzyme activity of 446.07 U/mg (Figure 7b). Thus, PoLDH expressed by Rosetta(DE3) was physically and biochemically highly similar to the actual protein and showed excellent enzyme activity. It is considered that the activity of our recombinant PoLDH may vary depending on the expressed host and strain. Compared to the reported study so far, the activity of our recombinant PoLDH was higher than that of *P. falciparum* LDH (PfLDH) and *P. vivax* LDH (PvLDH) expressed from BL21(DE3) at 266.0 U/mg and 236.6 U/mg, respectively [39]. However, it was slightly lower than the activity of recombinant *P. knowlesi* LDH (PkLDH) expressed from the same strain, which was 475.6 U/mg [40].

**Figure 8:**
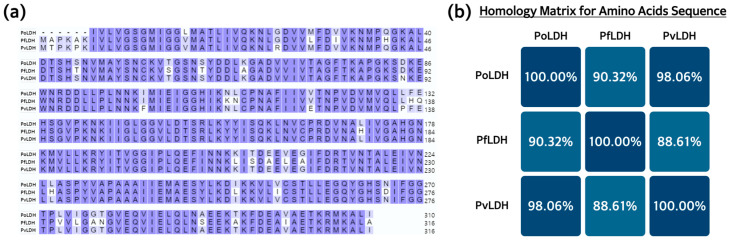
Amino acid sequence homology analysis of PoLDH, PfLDH, and PvLDH. (**a**) The amino acid sequences of PoLDH, PfLDH, and PvLDH. PoLDH consists of 310 amino acids, while PfLDH and PvLDH consist of 316 amino acids each. When the same amino acid exists at the same position, it is highlighted in purple, and the homology was analyzed using the UniProt homology analysis tool. (**b**) The homology matrix for PoLDH, PfLDH, and PvLDH. PoLDH showed 90.32% homology with PfLDH and 98.06% homology with PvLDH.

We expect that the recombinant PoLDH from this study could serve as a valuable tool for the specific diagnosis or treatment of malaria caused by *P. ovale*. We conducted an analysis of amino acid sequence homology between PoLDH and PfLDH as well as PvLDH using the UniProt database (Figure 8a). The results showed that PoLDH exhibited 90.32% sequence homology with PfLDH (UniProt ID: Q27743) and 98.06% sequence homology with PvLDH (UniProt ID: G5D073) (Figure 8b). While it is commonly thought that there is high homology among LDHs from species that produce dormant forms, this provides sufficient information to distinguish between them.

## 4. Conclusions

Our study results confirmed that the optimal conditions for PoLDH overexpression in *E. coli* included an IPTG concentration of 0.1 mM, a temperature of 18 °C, and an induction time of 16 h. Furthermore, we optimized the purification conditions of PoLDH recombinant proteins by varying the imidazole concentration using the optimal strain for expression, Rosetta(DE3). PoLDH and non-specific proteins in the elution fraction showed a purity of 99.9% when the washing buffer contained 40 mM imidazole. Rosetta(DE3) achieved the highest yield at 9.5 mg/L compared with that of the other two *E. coli* strains. Tetramer formation and excellent enzyme activity were confirmed using the purified PoLDH protein. Therefore, cloning, expression, and purification of PoLDH (a eukaryotic protein) were successfully achieved using an *E. coli* expression system, reproducing the original structure and function of the protein. To the best of our knowledge, this study is the first investigation to optimize the isolation and purification of PoLDH. We produced a large amount of soluble PoLDH, which is expected to contribute to research on malaria caused by *P. ovale*. In particular, the findings will be instrumental in the field of malaria diagnosis and the discovery of new therapeutics for the disease. Our findings can be used to develop diagnostic technologies for *P. ovale*-specific malaria targeting PoLDH. Furthermore, the study findings will be useful in discovering drug candidates that selectively inhibit PoLDH.

## Data Availability

Data are contained within the article.

## References

[1] Fikadu M., Ashenafi E. (2023). Malaria: An Overview. Infect. Drug Resist..

[2] World Health Organization (2023). World Malaria Report 2023.

[3] Phillips M.A., Burrows J.N., Manyando C., van Huijsduijnen R.H., Van Voorhis W.C., Wells T.N.C. (2017). Malaria. Nat. Rev. Dis. Primers.

[4] Cowman A.F., Healer J., Marapana D., Marsh K. (2016). Malaria: Biology and Disease. Cell.

[5] Garrido-Cardenas J.A., González-Cerón L., García-Maroto F., Cebrián-Carmona J., Manzano-Agugliaro F., Mesa-Valle C.M. (2023). Analysis of Fifty Years of Severe Malaria Worldwide Research. Pathogens.

[6] Venugopal K., Hentzschel F., Valkiūnas G., Marti M. (2020). *Plasmodium* Asexual Growth and Sexual Development in the Haematopoietic Niche of the Host. Nat. Rev. Microbiol..

[7] Sato S. (2021). *Plasmodium*—A Brief Introduction to the Parasites Causing Human Malaria and Their Basic Biology. J. Physiol. Anthropol..

[8] Veletzky L., Groger M., Lagler H., Walochnik J., Auer H., Fuehrer H.P., Ramharter M. (2018). Molecular Evidence for Relapse of an Imported *Plasmodium ovale wallikeri* Infection. Malar. J..

[9] Kotepui M., Masangkay F.R., Kotepui K.U., Milanez G.D.J. (2020). Misidentification of *Plasmodium ovale* as *Plasmodium vivax* Malaria by a Microscopic Method: A Meta-Analysis of Confirmed *P. ovale* Cases. Sci. Rep..

[10] White N.J. (2004). Antimalarial Drug Resistance. J. Clin. Investig..

[11] World Health Organization (2005). Susceptibility of Plasmodium falciparum to Antimalarial Drugs: Report on Global Monitoring: 1996–2004.

[12] McKeage K., Scott L.J. (2003). Atovaquone/Proguanil. Drugs.

[13] Wångdahl A., Sondén K., Wyss K., Stenström C., Björklund D., Zhang J., Askling H.H., Carlander C., Hellgren U., Färnert A. (2022). Relapse of *Plasmodium vivax* and *Plasmodium ovale* Malaria with and without Primaquine Treatment in a Nonendemic Area. Clin. Infect. Dis..

[14] Haston J.C., Hwang J., Tan K.R. (2019). Guidance for Using Tafenoquine for Prevention and Antirelapse Therapy for Malaria—United States, 2019. MMWR Morb. Mortal. Wkly. Rep..

[15] Kotepui M., Kotepui K.U., Milanez G.D., Masangkay F.R. (2020). Severity and Mortality of Severe *Plasmodium ovale* Infection: A Systematic Review and Meta-Analysis. PLoS ONE.

[16] Groger M., Fischer H.S., Veletzky L., Lalremruata A., Ramharter M. (2017). A Systematic Review of the Clinical Presentation, Treatment and Relapse Characteristics of Human *Plasmodium ovale* Malaria. Malar. J..

[17] Fuehrer H.P., Campino S., Sutherland C.J. (2022). The Primate Malaria Parasites *Plasmodium malariae*, *Plasmodium brasilianum* and *Plasmodium ovale* spp.: Genomic Insights into Distribution, Dispersal and Host Transitions. Malar. J..

[18] Sitali L., Miller J.M., Mwenda M.C., Bridges D.J., Hawela M.B., Hamainza B., Chizema-Kawesha E., Eisele T.P., Chipeta J., Lindtjørn B. (2019). Distribution of *Plasmodium* Species and Assessment of Performance of Diagnostic Tools Used During a Malaria Survey in Southern and Western Provinces of Zambia. Malar. J..

[19] Gibson L.E., Markwalter C.F., Kimmel D.W., Mudenda L., Mbambara S., Thuma P.E., Wright D.W. (2017). *Plasmodium falciparum* HRP2 ELISA for Analysis of Dried Blood Spot Samples in Rural Zambia. Malar. J..

[20] Priest J.W., Plucinski M.M., Huber C.S., Rogier E., Mao B., Gregory C.J., Candrinho B., Colborn J., Barnwell J.W. (2018). Specificity of the IgG Antibody Response to *Plasmodium falciparum*, *Plasmodium vivax*, *Plasmodium malariae*, and *Plasmodium ovale* MSP119 Subunit Proteins in Multiplexed Serologic Assays. Malar. J..

[21] Kim Y.J., Choi J.W. (2022). Enzyme-Linked Aptamer-Based Sandwich Assay (ELASA) for Detecting *Plasmodium falciparum* Lactate Dehydrogenase, a Malarial Biomarker. RSC Adv..

[22] Tang J., Tang F., Zhu H., Lu F., Xu S., Cao Y., Gu Y., He X., Zhou H., Zhu G. (2019). Assessment of False Negative Rates of Lactate Dehydrogenase-Based Malaria Rapid Diagnostic Tests for *Plasmodium ovale* Detection. PLoS Negl. Trop. Dis..

[23] Kori L.D., Valecha N., Anvikar A.R. (2020). Glutamate Dehydrogenase: A Novel Candidate to Diagnose *Plasmodium falciparum* through Rapid Diagnostic Test in Blood Specimen from Fever Patients. Sci. Rep..

[24] Mathema V.B., Na-Bangchang K. (2015). A Brief Review on Biomarkers and Proteomic Approach for Malaria Research. Asian Pac. J. Trop. Med..

[25] Plucinski M.M., McElroy P.D., Dimbu P.R., Fortes F., Nace D., Halsey E.S., Rogier E. (2019). Clearance Dynamics of Lactate Dehydrogenase and Aldolase Following Antimalarial Treatment for *Plasmodium falciparum* Infection. Parasit. Vectors.

[26] Zhou Y., Qi M., Yang M. (2022). Current Status and Future Perspectives of Lactate Dehydrogenase Detection and Medical Implications: A Review. Biosensors.

[27] Rosano G.L., Ceccarelli E.A. (2014). Recombinant Protein Expression in *Escherichia coli*: Advances and Challenges. Front. Microbiol..

[28] Rosano G.L., Morales E.S., Ceccarelli E.A. (2019). New Tools for Recombinant Protein Production in *Escherichia coli*: A 5-Year Update. Protein Sci..

[29] Hayat S.M.G., Farahani N., Golichenari B., Sahebkar A. (2018). Recombinant Protein Expression in *Escherichia coli* (*E.coli*): What We Need to Know. Curr. Pharm. Des..

[30] Turgut-Balik D., Akbulut E., Shoemark D.K., Celik V., Moreton K.M., Sessions R.B., Holbrook J.J., Brady R.L. (2004). Cloning, Sequence and Expression of the Lactate Dehydrogenase Gene from the Human Malaria Parasite, *Plasmodium vivax*. Biotechnol. Lett..

[31] Shin H.I., Kim J.Y., Lee W.J., Sohn Y., Lee S.W., Kang Y.J., Lee H.W. (2013). Polymorphism of the Parasite Lactate Dehydrogenase Gene from *Plasmodium vivax* Korean Isolates. Malar. J..

[32] Jeon W., Lee S., Manjunatha D.H., Ban C. (2013). A Colorimetric Aptasensor for the Diagnosis of Malaria Based on Cationic Polymers and Gold Nanoparticles. Anal. Biochem..

[33] Sousa L.P., Mariuba L.A.M., Holanda R.J., Pimentel J.P., Almeida M.E.M., Chaves Y.O., Borges D., Lima E., Crainey J.L., Orlandi P.P. (2014). A Novel Polyclonal Antibody-Based Sandwich ELISA for Detection of *Plasmodium vivax* Developed from Two Lactate Dehydrogenase Protein Segments. BMC Infect. Dis..

[34] Tegel H., Tourle S., Ottosson J., Persson A. (2010). Increased Levels of Recombinant Human Proteins with the *Escherichia coli* Strain Rosetta(DE3). Protein Expr. Purif..

[35] Kang Y., Son M.S., Hoang T.T. (2007). One Step Engineering of T7-Expression Strains for Protein Production: Increasing the Host-Range of the T7-Expression System. Protein Expr. Purif..

[36] Kim Y.J., Shin J.S., Lee K.W., Eom H.J., Jo B.G., Lee J.W., Kim J.H., Kim S.Y., Kang J.H., Choi J.W. (2023). Expression, Purification, and Characterization of *Plasmodium vivax* Lactate Dehydrogenase from Bacteria without Codon Optimization. Int. J. Mol. Sci..

[37] Hebditch M., Carballo-Amador M.A., Charonis S., Curtis R., Warwicker J. (2017). Protein-Sol: A web tool for predicting protein solubility from sequence. Bioinformatics.

[38] Oeller M., Kang R., Bell R., Ausserwöger H., Sormanni P., Vendruscolo M. (2023). Sequence-based prediction of pH-dependent protein solubility using CamSol. Brief Bioinform..

[39] Keluskar P., Ingle S. (2012). Ethnopharmacology Guided Screening of Traditional Indian Herbs for Selective Inhibition of *Plasmodium* Specific Lactate Dehydrogenase. J. Ethnopharmacol..

[40] Salim N.O., Fuad F.A.A., Khairuddin F., Seman W.M.K.W., Jonet M.A. (2021). Purifying and Characterizing Bacterially Expressed Soluble Lactate Dehydrogenase from *Plasmodium knowlesi* for the Development of Anti-Malarial Drugs. Molecules.

